# A study of analyzing longitudinal dynamic behavior of a double-rod system with longitudinal nonlinear supports

**DOI:** 10.1038/s41598-024-58986-9

**Published:** 2024-04-07

**Authors:** Yuhao Zhao, Haijian Cui

**Affiliations:** 1 Key Laboratory of Advanced Manufacturing Technology of the Ministry of Education, Guizhou University, Guiyang, 550025 People’s Republic of China; 2 Wuhan Second Ship Design and Research Institute, Wuhan, 430064 People’s Republic of China

**Keywords:** Nonlinear vibration, Double-rod system, Nonlinear supports, Galerkin method, Energy science and technology, Engineering

## Abstract

In engineering, shafting systems are typically subjected to longitudinal vibration excitations, which may result in unwanted vibration. To study the control of longitudinal vibration in shafting systems, they can be simplified to rod structures. Currently, engineers have attempted to apply the nonlinear principle to design nonlinear supports to control the vibration of flexible structures. However, the flexible structures referenced in the literature are usually composed of a single component, which limits the application of nonlinear supports to more complex structures. To explore the potential application of nonlinear supports in marine engineering, this work introduces a longitudinal vibration prediction model for a double-rod system equipped with longitudinal nonlinear supports. The generalized Hamilton principle is used to derive the governing equations for the double-rod system with longitudinal nonlinear supports. The longitudinal vibration responses of the double-rod system are numerically solved using the Galerkin truncation method. The numerical results confirm that a 1-term truncation number guarantees the stability of the longitudinal vibration prediction model. Under certain conditions, the longitudinal vibration responses are significantly affected by longitudinal nonlinear supports. It is recommended to install longitudinal nonlinear supports on both Rod 1 and Rod 2 simultaneously to suppress vibration in the first two main resonance orders. With reasonable excitations, the vibration state and magnitudes of the double-rod system can be effectively controlled by adjusting the longitudinal nonlinear supports. Complex longitudinal vibration responses are more readily induced by altering the parameters of the longitudinal nonlinear support installed on Rod 1. Choosing appropriate parameters for the nonlinear supports on Rod 1 and Rod 2 positively contributes to the reduction of vibration in the double-rod system.

## Introduction

In various engineering fields, complex systems comprising basic engineering units—such as rods, beams, plates, shells, and others—are inevitably subject to vibration excitations caused by power machinery. In most cases, these excitations will induce external vibrations in complex systems. Unfortunately, prolonged exposure to strong vibration excitations can lead to several problems, including structural fatigue and fractures. Therefore, controlling vibrations in complex systems is vital to maintain their safety.

In marine engineering, shafting systems generally experience longitudinal vibration excitations introduced by the propeller, with excitation frequencies that correlate with the propeller’s rotational speed. Moreover, when analyzing the longitudinal vibration of shafting systems, they are often simplified to a system of multiple rods, while thrust bearings are represented as longitudinal support springs. Against this engineering background, understanding the longitudinal vibration characteristics of rod systems is critical for controlling vibrations in shafting systems. Numerous scholars have investigated the linear longitudinal vibration characteristics of rod systems, including rod mode shapes^1^, characteristic equations of rods^2^, analytical solutions for rods^3^, comparative studies of rod theories^4^, and rapid solution methods for rods^5^. With the advancement of nonlinear vibration theory, Cao and Tucker^6^ examined the nonlinear dynamics of elastic rods using Cosserat theory. Wang and Li^7^ developed a vibration model consisting of two rods and a clearance joint and discussed the model’s nonlinear dynamics. Andrianov et al.^8^ introduced a model of continuous rods with microstructure and investigated its nonlinear vibrations. Wang et al.^9^ studied the nonlinear vibration responses of a rod-fastening rotor based on the internal damping effect. Malara et al.^10^ considered the fractional derivative element and researched the nonlinear vibration of rods with variable cross-sections. Liang et al.^11–13^ developed the spectral element method, the transfer matrix method, and other robust approaches to systematically study complex vibrations in fluid-conveying pipes, contributing to the advancement of vibration prediction methods for elastic structures. With the increasing demand for nonlinear vibration control, scholars have attempted to utilize nonlinear principles to design various nonlinear supports^14–17^ Engineers then installed these supports into elastic structures to assess the feasibility of controlling vibrations using nonlinear supports. Santo et al.^18^ constructed a vibration prediction model for a vibrating rod with nonlinear springs and studied its multimodal behavior. Hao et al.^19^ designed a nonlinear vibration isolator and incorporated it into a flexible plate. They thoroughly investigated the isolator’s effectiveness in isolating the plate’s vibrations, advancing the application of nonlinear controls in flexible structures. Subsequently, many researchers focused on beam vibration control using nonlinear supports. Senalp et al.^20^ equipped a finite-length Euler–Bernoulli beam with linear and nonlinear foundations to examine its nonlinear dynamic responses. Ghayesh et al.^21–23^ considered supports exhibiting cubic stiffness characteristics and applied this type of nonlinear support at the internal boundaries of flexible beams. They explored the nonlinear vibrations, stability, and bifurcations of the system, facilitating the implementation of nonlinear stiffness in engineering. Wang and Fang^24^ analyzed the nonlinear vibrations of a flexible beam with nonlinear supports at both ends. Rodrigues et al.^25^ investigated the dynamic behavior of a beam on nonlinear elastic foundations. Mao et al.^26^ used the multiple scales method to study the nonlinear vibrations of flexible beams with nonlinear boundaries. Ding et al.^27,28^ introduced an adjustable nonlinear support design and implemented it in flexible and curved beams, proposing a novel approach to vibration control. Basta et al.^29^ utilized nonlinear energy sinks to create a metamaterial for a rotating cantilever beam and probed its nonlinear dynamics. Zhao et al.^30–32^ placed nonlinear supports in single and double-beam systems and systematically examined how the supports’ parameters affected the beams’ vibration responses. Han et al.^33^ applied the Lagrange method (LM) to assess the nonlinear dynamics of a beam with multiple nonlinear supports. The aforementioned studies primarily concentrate on the nonlinear vibration responses of flexible structures with nonlinear supports. However, these studies typically involve structures composed of a single element, which limits the application of nonlinear supports in more complex systems.

Considering marine engineering, the shaft systems suffer the axial force introduced by the propeller, which causes longitudinal vibration of the shafting systems, where such complex shafting systems are typically composed of several shaft segments. In analyzing the longitudinal vibration of some shafting systems, they can be simplified as the multiple-rod system connected through coupling elements. To explore the potential application of nonlinear supports in the vibration control of marine engineering, this work proposes a longitudinal vibration prediction model of a double-rod system with longitudinal nonlinear supports. Governing equations of the double-rod system with longitudinal nonlinear supports are derived by using the generalized Hamilton principle. Then, the double-rod system’s longitudinal vibration responses are numerically solved through the Galerkin truncation method (GTM). Based on the correct numerical results, longitudinal vibration responses of the double-rod system impacted by longitudinal nonlinear supports are studied.

## Theoretical formulations

### Model description

**Figure 1:**
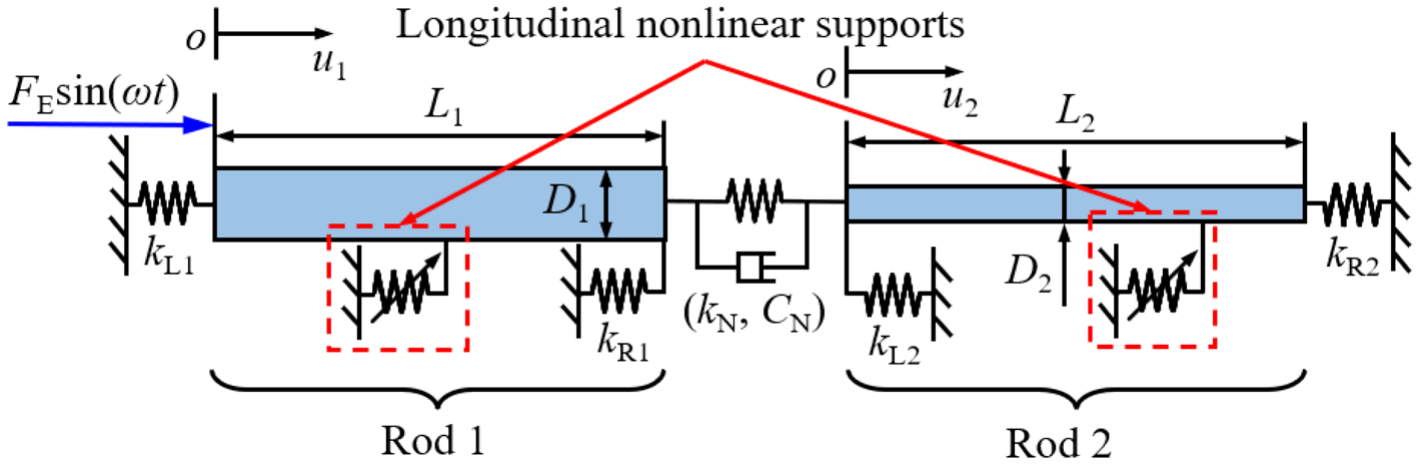
The schematic diagram of a double-rod system with longitudinal nonlinear supports.

In marine engineering, rod structures can be employed to simply shafting systems in analyzing their longitudinal vibration characteristics, where shafting systems are typically simplified as the vibration system consists of multiple rods, whereas thrust bearings in shafting systems are simplified to longitudinal support springs. For most occasions, shafting systems are generally subjected to longitudinal vibration excitations introduced by the propeller, where excitation frequencies of such vibration excitations are related to the rotation speed of the propeller. Generally, vibration excitation is acting on one component of the shafting system and transfer from couplers to other components of the system. Against this background, Fig. 1 gives the schematic diagram of a double-rod system with two longitudinal nonlinear supports. Rods of the system are marked as Rod 1 and Rod 2 to simplify the derivation, where *u*_1_(*x*_1_,*t*) and *u*_2_(*x*_2_,*t*) are the longitudinal vibration displacements of rods. Rods in the vibration system are connected through the linear coupling element, which is simplified as the coupling stiffness and coupling viscous damping. Longitudinal supporting springs are installed at the boundaries of the double-rod system. Various boundary conditions of the double-rod system can be simulated by changing the stiffness of the supporting springs.

Longitudinal nonlinear supports are installed at Rod 1 and Rod 2, respectively. In this work, each nonlinear support presents the cubic stiffness character, where *k*_N1_ and *k*_N2_ are defined as their longitudinal nonlinear stiffness. *x*_N1_ and *x*_N2_ are the specific locations of longitudinal nonlinear supports at the double-rod system. Furthermore, the harmonic excitation is located at the left boundary of the double-rod system. Table 1 presents definitions of the parameters belonging to the vibration system.

**Table 1:** Definitions of structural parameters belonging to the double-rod system.

Parameters	Symbol	Unit
Elastic modulus of rods	*E*_1_/*E*_2_	Pa
Density of rods	*ρ*_1_/*ρ*_2_	kg/m^3^
Length of rods	*L*_1_/*L*_2_	m
Diameter of rods	*D*_1_/*D*_2_	m
Section area of rods	*S*_1_/*S*_2_	m^2^
Coupling linear stiffness	*k*_E_	N/m
Coupling viscous damping	*C*_E_	Ns/m
Longitudinal supporting stiffness	*k*_L1_/*k*_R1_/*k*_L2_/*k*_R2_	N/m

### Equations derivation

This section derives governing equations of the double-rod system with longitudinal nonlinear supports. Before the derivation process, it is the basement to derive the energy form of the double-rod system with longitudinal nonlinear supports. Firstly, the kinetic energy of the double-rod system with longitudinal nonlinear supports is derived as,

1 $$T_{{{\text{System}}}} = T_{{{\text{Rod1}}}} + T_{{{\text{Rod2}}}}$$

*T*_Rod1_ and *T*_Rod2_ are the kinetic energy of Rod 1 and Rod 2. The potential energy of the double-rod system with longitudinal nonlinear supports is derived as,

2 $$V_{{{\text{System}}}} = V_{{{\text{Rod1}}}} + V_{{{\text{Rod2}}}} + V_{{{\text{Boundary1}}}} + V_{{{\text{Boundary2}}}} + V_{{{\text{LE}}}} + V_{{{\text{LN1}}}} + V_{{{\text{LN2}}}}$$

*V*_Rod1_ and *V*_Rod2_ are the potential of Rod 1 and Rod 2. *V*_Boundary1_ and *V*_Boundary2_ are the potential energy of longitudinal supporting springs belonging to Rod 1 and Rod 2. *V*_LE_ is the potential energy of the linear coupling element. *V*_LN1_ and *V*_LN2_ are the potential energy of longitudinal nonlinear supports.

Furthermore, the virtual work done by the coupling viscous damping and harmonic excitation is derived as,

3 $$\delta W_{{{\text{System}}}} = \delta W_{{{\text{LE}}}} + \delta W_{{\text{E}}}$$

*δW*_LE_ is the virtual work done by the linear coupling element. *δW*_E_ is the virtual work done by the harmonic excitation. The specific expressions of each term in Eqs. (1) to (3) are listed in Appendix A.

Then, employing the generalized Hamilton principle, namely,

4 $$\int_{{t_{1} }}^{{t_{2} }} {\delta \left( {T_{{{\text{System}}}} - V_{{{\text{System}}}} } \right){\text{d}}t} + \int_{{t_{1} }}^{{t_{2} }} {\delta W_{{{\text{System}}}} {\text{d}}t} = 0$$

On this basis, governing equations of the double-rod system with longitudinal nonlinear supports are derived by using the variational principle,

5a $$\begin{gathered} \rho_{1} S_{1} \frac{{\partial^{2} u_{1} }}{{\partial t^{2} }} - E_{1} S_{1} \frac{{\partial^{2} u_{1} }}{{\partial x_{1}^{2} }} + \delta \left( {x_{1} - x_{{{\text{N1}}}} } \right)k_{{{\text{N1}}}} u_{1}^{3} + \delta \left( {x_{{1}} } \right)F_{{\text{E}}} \sin \left( {\omega t} \right) \hfill \\ \begin{array}{*{20}c} {} \\ \end{array} + \delta \left( {x_{1} - L_{1} } \right)\delta \left( {x_{2} } \right)\left[ {k_{{\text{E}}} \left( {u_{1} - u_{2} } \right) + C_{{\text{E}}} \left( {\frac{{\partial u_{1} }}{\partial t} - \frac{{\partial u_{2} }}{\partial t}} \right)} \right] = 0 \hfill \\ \end{gathered}$$

and

5b $$\begin{aligned} & \rho_{2} S_{2} \frac{{\partial^{2} u_{2} }}{{\partial t^{2} }} - E_{2} S_{2} \frac{{\partial^{2} u_{2} }}{{\partial x_{2}^{2} }} + \delta \left( {x_{2} - x_{{{\text{N2}}}} } \right)k_{{{\text{N2}}}} u_{2}^{3} \\ & \;\; + \delta \left( {x_{1} - L_{1} } \right)\delta \left( {x_{2} } \right)\left[ {k_{{\text{E}}} \left( {u_{2} - u_{1} } \right) + C_{{\text{E}}} \left( {\frac{{\partial u_{2} }}{\partial t} - \frac{{\partial u_{1} }}{\partial t}} \right)} \right] = 0 \\ \end{aligned}$$

Equation (5a) is the vibration-governing equation of Rod 1 while Eq. (5b) is the vibration-governing equation of Rod 2. Meanwhile, boundary-governing equations of the double-rod system are derived as,

6a $$\left\{ \begin{gathered} k_{{{\text{L1}}}} u_{1} - E_{1} S_{1} \frac{{\partial u_{1} }}{{\partial x_{1} }} = 0\begin{array}{*{20}c} , \\ \end{array} \begin{array}{*{20}c} {} \\ \end{array} x_{1} = 0 \hfill \\ k_{{{\text{R1}}}} u_{1} + E_{1} S_{1} \frac{{\partial u_{1} }}{{\partial x_{1} }} = 0\begin{array}{*{20}c} , \\ \end{array} \begin{array}{*{20}c} {} \\ \end{array} x_{1} = L_{1} \hfill \\ \end{gathered} \right.$$

and

6b $$\left\{ \begin{gathered} k_{{{\text{L2}}}} u_{2} - E_{2} S_{2} \frac{{\partial u_{2} }}{{\partial x_{2} }} = 0\begin{array}{*{20}c} , \\ \end{array} \begin{array}{*{20}c} {} \\ \end{array} x_{2} = 0 \hfill \\ k_{{{\text{R2}}}} u_{2} + E_{2} S_{2} \frac{{\partial u_{2} }}{{\partial x_{2} }} = 0\begin{array}{*{20}c} , \\ \end{array} \begin{array}{*{20}c} {} \\ \end{array} x_{2} = L_{2} \hfill \\ \end{gathered} \right.$$

Equation (6a) is the boundary-governing equation of Rod 1 while Eq. (6b) is the boundary-governing equation of Rod 2. The specific derivation process of the double-rod system with longitudinal nonlinear supports related to Eq. (4) is listed in Appendix B.

### Procedure of solution

Based on the governing equations derived in "Equations derivation" section longitudinal vibration responses of the double-rod system with longitudinal nonlinear supports can be obtained by solving its governing equations. In this work, the modal information of the double-rod system can be easily obtained. Considering the characteristics of GTM, the modal information of double-rod system provides the convenience for using the Galerkin truncation method to predict longitudinal dynamic behavior of a double-rod system with longitudinal nonlinear supports. Thus, this section concentrates on the solution procedure of the double-rod system with longitudinal nonlinear supports by using the Galerkin truncation method (GTM).

Before solving the governing equations derived in "Equations derivation" section it is necessary to expand the longitudinal vibration displacements of the double-rod system. In this work, the modal superposition method is applied to expand the above displacements, namely,

7a $$u_{1} \left( {x_{1} ,t} \right) = \sum\limits_{n = 1}^{{\text{N}}} {\varphi_{1n} \left( {x_{1} } \right)q_{1n} \left( t \right)}$$

and

7b $$u_{2} \left( {x_{2} ,t} \right) = \sum\limits_{m = 1}^{{\text{M}}} {\varphi_{2m} \left( {x_{2} } \right)q_{2m} \left( t \right)}$$

*φ*_1*n*_(*x*_1_) and *φ*_2*m*_(*x*_2_) are the mode functions of rods. *q*_1*n*_(*t*) and *q*_2*m*_(*t*) are the undetermined time coefficients. N and M are the max values of *n* and *m*. Substituting Eq. (7) into Eq. (5) and using the Galerkin condition, vibration-governing equations of rods are dispersed as,

8a $$\begin{aligned} & \int_{0}^{{L_{1} }} {\left( {\rho_{1} S_{1} \sum\limits_{n = 1}^{{\text{N}}} {\varphi_{1n} \frac{{{\text{d}}^{2} q_{1n} }}{{{\text{d}}t^{2} }}} - E_{1} S_{1} \sum\limits_{n = 1}^{{\text{N}}} {\frac{{{\text{d}}^{2} \varphi_{1n} }}{{{\text{d}}x_{1}^{2} }}q_{1n} } } \right)\psi_{1i} \left( {x_{1} } \right){\text{d}}x_{1} } \\ & \;\; + F_{{\text{E}}} \sin \left( {\omega t} \right)\left[ {\sum\limits_{n = 1}^{{\text{N}}} {\varphi_{1n} \left( 0 \right)q_{1n} } } \right]\psi_{1i} \left( 0 \right) \\ & \;\; + k_{{\text{E}}} \left[ {\sum\limits_{n = 1}^{{\text{N}}} {\varphi_{1n} \left( {L_{1} } \right)q_{1n} } - \sum\limits_{m = 1}^{{\text{M}}} {\varphi_{2m} \left( 0 \right)q_{2m} } } \right]\psi_{1i} \left( {L_{1} } \right) \\ & \;\; + C_{{\text{E}}} \left[ {\sum\limits_{n = 1}^{{\text{N}}} {\varphi_{1n} \left( {L_{1} } \right)\frac{{{\text{d}}q_{1n} }}{{{\text{d}}t}}} - \sum\limits_{m = 1}^{{\text{M}}} {\varphi_{2m} \left( 0 \right)\frac{{{\text{d}}q_{2m} }}{{{\text{d}}t}}} } \right]\psi_{1i} \left( {L_{1} } \right) \\ & \;\; + k_{{{\text{N1}}}} \left[ {\sum\limits_{n = 1}^{{\text{N}}} {\varphi_{1n} \left( {x_{{{\text{N1}}}} } \right)\frac{{{\text{d}}q_{2n} }}{{{\text{d}}t}}} } \right]^{3} \psi_{1i} \left( {x_{{{\text{N1}}}} } \right) = 0 \\ \end{aligned}$$

and

8b $$\begin{aligned} & \int_{0}^{{L_{2} }} {\left( {\rho_{2} S_{2} \sum\limits_{m = 1}^{{\text{M}}} {\varphi_{2m} \frac{{{\text{d}}^{2} q_{2m} }}{{{\text{d}}t^{2} }}} - E_{2} S_{2} \sum\limits_{m = 1}^{{\text{M}}} {\frac{{{\text{d}}^{2} \varphi_{2m} }}{{{\text{d}}x_{2}^{2} }}q_{2m} } } \right)\psi_{2j} \left( {x_{2} } \right){\text{d}}x_{2} } \\ & \;\; + k_{{\text{E}}} \left[ {\sum\limits_{m = 1}^{{\text{M}}} {\varphi_{2m} \left( 0 \right)q_{2m} } - \sum\limits_{n = 1}^{{\text{N}}} {\varphi_{1n} \left( {L_{1} } \right)q_{1n} } } \right]\psi_{2j} \left( 0 \right) \\ & \;\; + C_{{\text{E}}} \left[ {\sum\limits_{m = 1}^{{\text{M}}} {\varphi_{2m} \left( 0 \right)\frac{{{\text{d}}q_{2m} }}{{{\text{d}}t}}} - \sum\limits_{n = 1}^{{\text{N}}} {\varphi_{1n} \left( {L_{1} } \right)\frac{{{\text{d}}q_{1n} }}{{{\text{d}}t}}} } \right]\psi_{2j} \left( 0 \right) \\ & \;\; + k_{{{\text{N2}}}} \left[ {\sum\limits_{m = 1}^{{\text{M}}} {\varphi_{2m} \left( {x_{{{\text{N2}}}} } \right)\frac{{{\text{d}}q_{2m} }}{{{\text{d}}t}}} } \right]^{3} \psi_{2j} \left( {x_{{{\text{N2}}}} } \right) = 0 \\ \end{aligned}$$

The max value of *i* is N while that of *j* is M. Furthermore, *ψ*_1*i*_(*x*_1_) and *ψ*_2*j*_(*x*_2_) are the weight functions, meeting boundary-governing equations derived in "Equations derivation" section. In this work, the mode functions of each rod are selected as the weight functions. It can be easily observed that the solution procedure derived in this section is based on the modal information of rods, which is called the Galerkin truncation method. Then, longitudinal vibration responses of the double-rod system with longitudinal nonlinear supports are gained by applying numerical methods to solve Eqs. (8a) and (b). In this work, the Runge–Kutta method is chosen to solve the above equations.

## Numerical analysis and discussion

The solution procedure derived in "Theoretical formulations" section is realized in the simulation platform MATLAB, where the ODE45 is employed to numerically solve the residual equations of the double-rod system with longitudinal nonlinear supports. Longitudinal vibration responses of the double-rod system with longitudinal nonlinear supports are numerically obtained. At first, the validation of longitudinal vibration responses calculated by the GTM is studied. Then, the impact of longitudinal nonlinear supports on the longitudinal vibration responses of the double-rod system is discussed. In the process of calculating, the calculation domain is chosen as 2000 periods of harmonic excitation. Longitudinal vibration responses in the last 200 calculation periods are regarded as stable numerical results. Structural parameters of the system are listed as follows, *E*_1_ = *E*_2_ = 6.89 × 10^10^ Pa, *D*_1_ = 0.06 m, *D*_2_ = 0.04 m, *ρ*_1_ = *ρ*_2_ = 2800 kg/m^3^, *L*_1_ = *L*_2_ = 0.5 m, *k*_E_ = 10^4^ N/m, *C*_E_ = 2 Ns/m, *F*_E_ = 100 N, and *k*_L1_ = *k*_L2_ = *k*_R1_ = *k*_R2_ = 5 × 10^4^ N/m.

### Validity study

**Figure 2:**
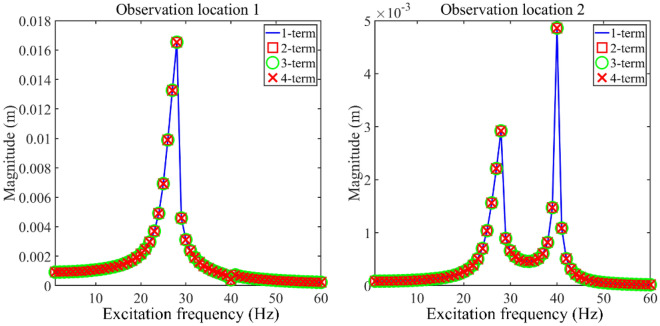
Longitudinal vibration responses under different truncation numbers.

**Figure 3:**
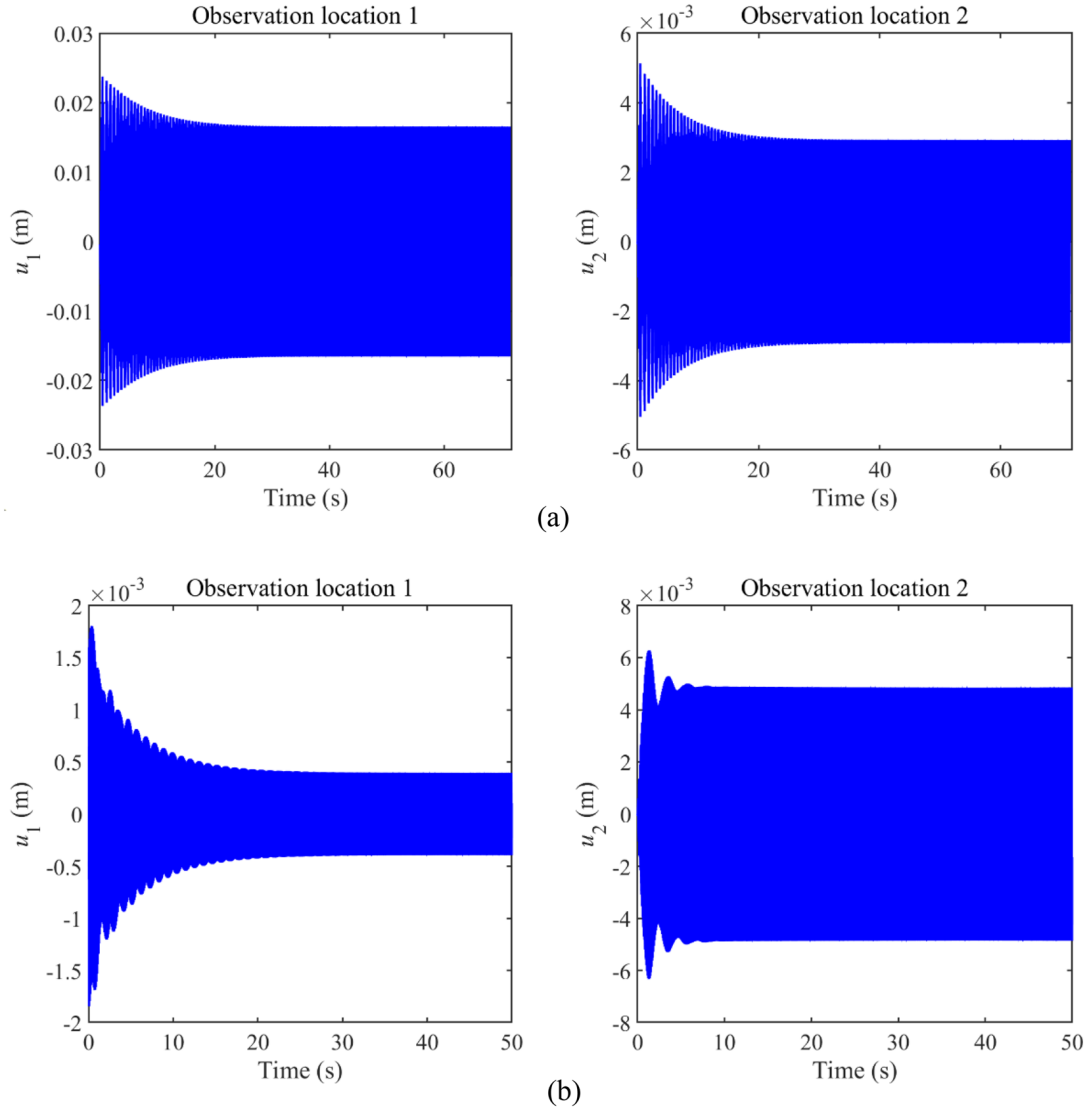
Time history of the longitudinal vibration responses under certain excitation frequencies.

This section studies the validity of longitudinal vibration responses of the double-rod system with longitudinal nonlinear supports. In this numerical example, the parameters of nonlinear supports are chosen as *k*_N1_ = 10^8^ N/m^3^ and *k*_N2_ = 10^8^ N/m^3^. Observation location 1 is *x*_1_ = *L*_1_ while observation location 2 is *x*_2_ = 0. Figure 2 gives the longitudinal vibration responses under different truncation numbers. From Fig. 2, it can be observed that longitudinal vibration responses remain stable under 1-term, 2-term, 3-term, and 4-term truncation numbers. The above phenomenon indicates that 1-term truncation number can guarantee the stability of the longitudinal vibration responses of the double-rod system with nonlinear supports. Thus, in the subsequent study, the 1-term truncation number is set to calculate longitudinal vibration responses of the double-rod system in the subsequent study. Figure 3 gives the time history of the vibration responses belonging to the double-rod system under certain excitation frequencies. It can be seen from Fig. 3 that longitudinal vibration responses fully remain convergence in the last 200 calculation periods, illustrating the rationality of selecting calculation periods in this work.

**Figure 4:**
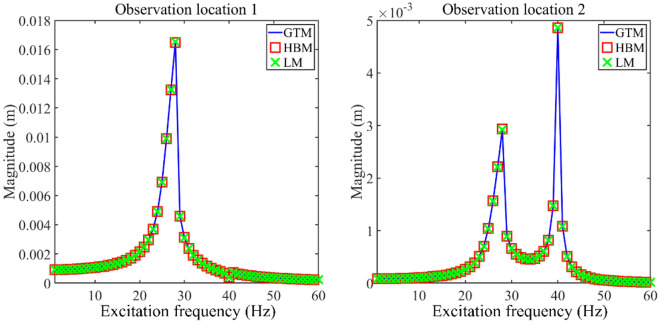
Longitudinal vibration responses gained by different methods.

Figure 4 gives longitudinal vibration responses gained by different methods, where the harmonic balance method (HBM) and Lagrange method (LM) are applied to verify the correctness of GTM in obtaining longitudinal vibration responses of the double-rod system. The detailed processes of LM and HBM are listed in Appendices C and D. Importantly, the solution process of LM is different from that of GTM. Meanwhile, the GTM calculates longitudinal dynamic behavior of the double-rod system with longitudinal nonlinear supports from the time domain while the HBM calculates those from the frequency domain. From Fig. 4, longitudinal vibration responses of the double-rod system with longitudinal nonlinear supports calculated by GTM fit smoothly with those gained by other methods. According to the above analysis, the correctness of GTM in calculating longitudinal vibration responses of the double-rod system with longitudinal nonlinear supports is verified.

### Longitudinal frequency responses impacted by longitudinal nonlinear supports

This work mainly concentrates on the longitudinal dynamic behavior of the double-rod system influenced by longitudinal nonlinear supports. In this section, the influence of parameters belonging to nonlinear supports mainly are systemically studied.

**Figure 5:**
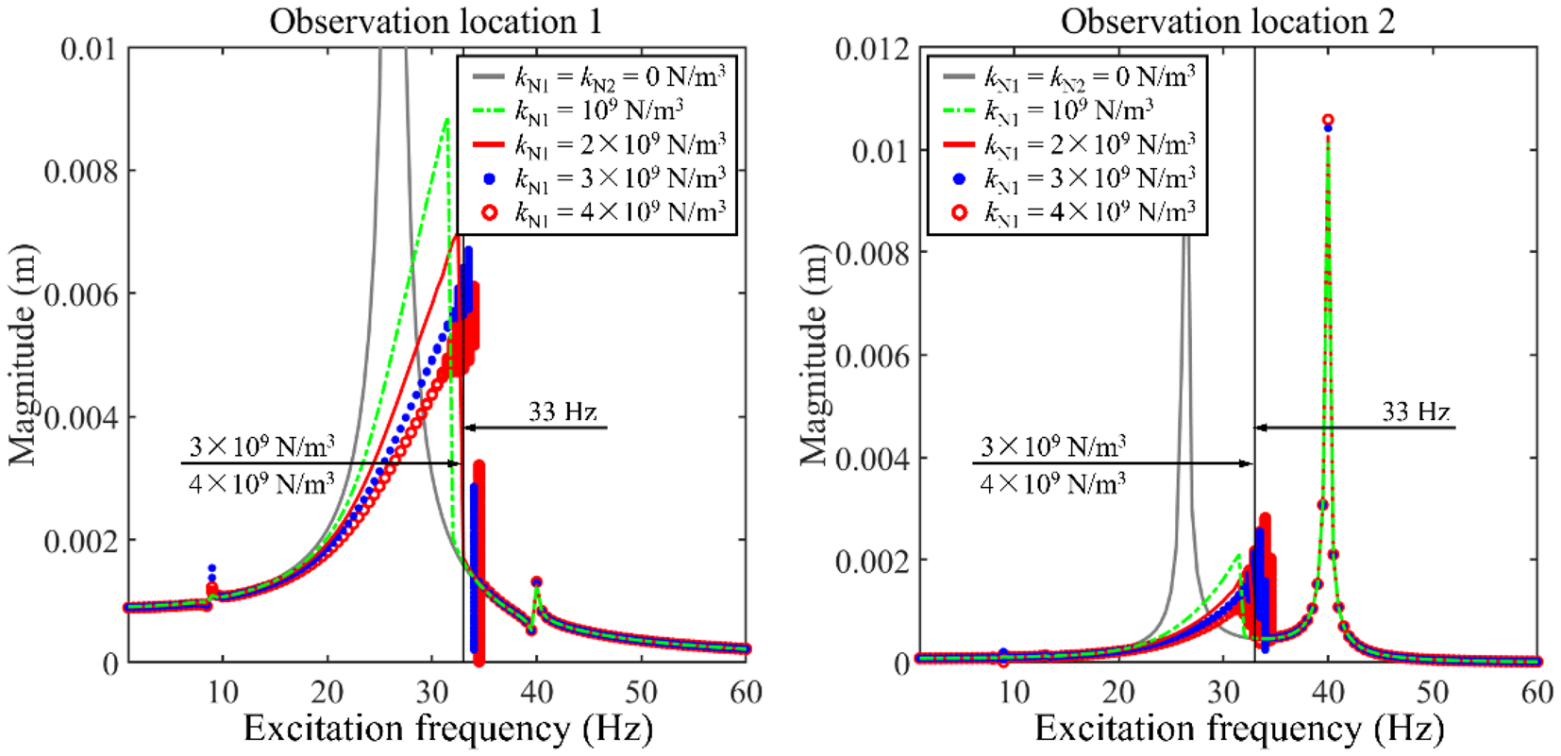
The impact of *k*_N1_ on longitudinal frequency responses.

Figure 5 gives the impact of *k*_N1_ on longitudinal frequency responses. In this numerical example, the longitudinal nonlinear support only installs on Rod 1. From Fig. 5, the parameter variation of longitudinal nonlinear support which is installed on Rod 1 impacts longitudinal vibration responses greatly for the 1^st^ main resonance area. In contrast, the variation of *k*_N1_ rarely impacts longitudinal vibration responses at the 2^nd^ main resonance area. With the growth of *k*_N1_, the 1^st^ main resonance area gradually shifts to a higher frequency area. Meanwhile, the existence of longitudinal nonlinear support suppresses the vibration level of the 1^st^ main resonance area for both rods. The magnitude jumping phenomenon can be observed due to the existence of *k*_N1_. The reason for the above phenomenon is that vibration magnitudes of the double-rod system at resonance areas stay in a significant level. According to the vibration theory, as the excitation frequency runs away from the resonance areas of the double-rod system, vibration magnitude of double-rod system decreases sharply. Due to the existence of supporting nonlinear stiffness, magnitude-frequency response curves are no longer symmetric. Thus, magnitude jumping phenomenon can be clearly observed. In addition, the 1/3 sub-harmonic resonance is motivated due to the existence of longitudinal nonlinear support installed in Rod 1, where the fundamental natural frequency of Rod 1 is 25.3 Hz. From the aspect of vibration states, under *k*_N1_ = 3 × 10^9^ N/m^3^ and *k*_N1_ = 4 × 10^9^ N/m^3^, longitudinal vibration responses present multiple continuous amplitudes. It should be noticed that the vibration state of longitudinal vibration responses that have multiple continuous amplitudes is different from other longitudinal vibration responses. According to the above phenomenon, the longitudinal nonlinear support that is installed on Rod 1 converts the vibration state of the double-rod system under certain values of *k*_N1_.

**Figure 6:**
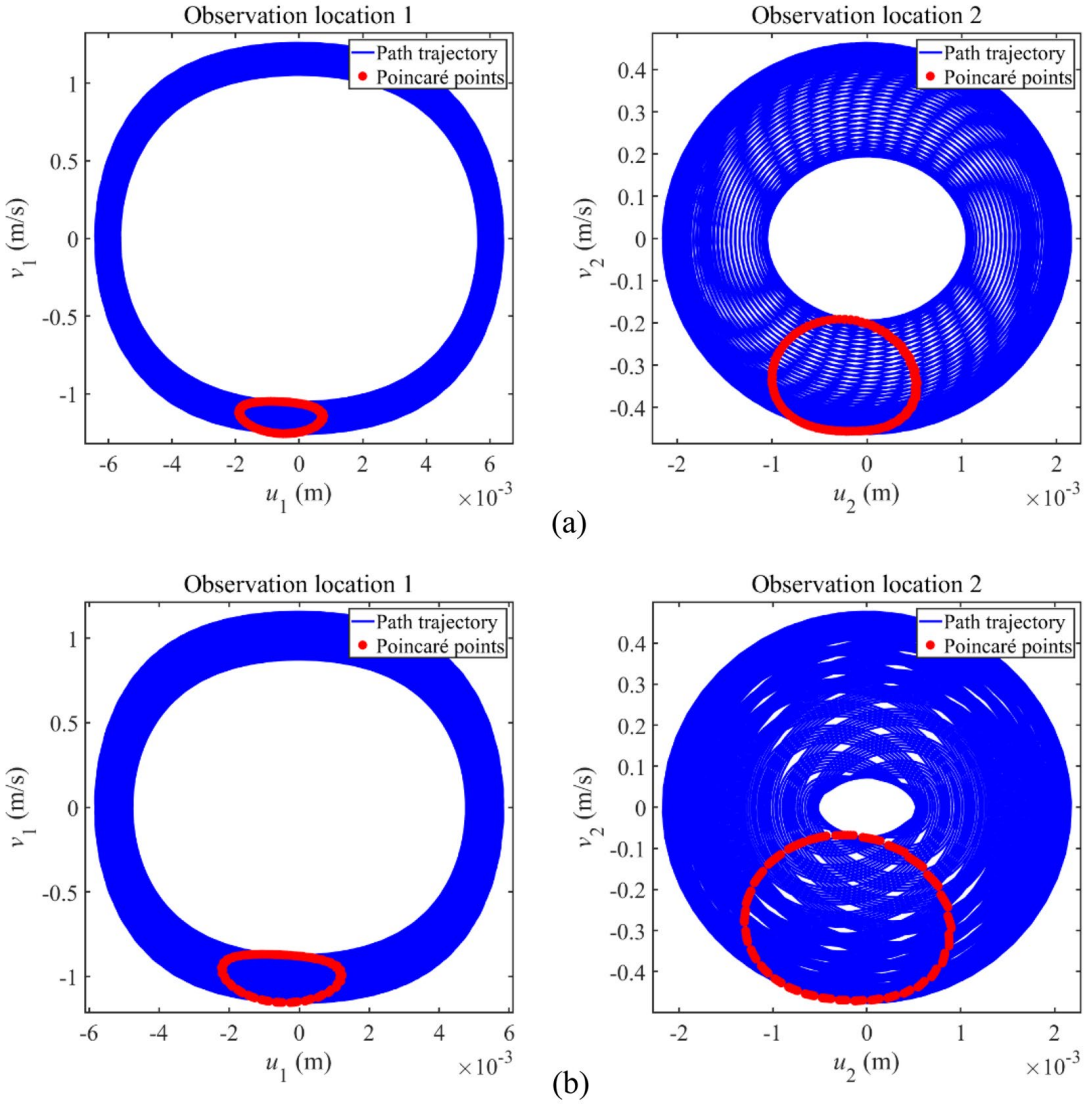
Phase trajectory and Poincaré points of complex longitudinal vibration responses.

To study the vibration state of complex longitudinal vibration responses, Fig. 6 gives the phase trajectory and Poincaré points of complex longitudinal vibration responses. In plotting Poincaré points, a constant phase *t*_0_ is selected as the initial phase while external excitation period (*t* = *t*_0_, *t* = *t*_0_ + *T*_E_, *t* = *t*_0_ + 2*T*_E_ …) is selected as the variation period to plot steady-state response points in phase diagrams. The steady-state response points gained through the above process are exactly Poincaré points. Furthermore, *v*_1_ and *v*_2_ are defined as the velocity of Rod 1 and Rod 2, respectively. From each subfigure in Fig. 6, the phase trajectory remains stable and Poincaré points form an entire closed curve. It can be concluded that complex longitudinal vibration responses that appeared in Fig. 5 stay in the quasi-periodic vibration state.

**Figure 7:**
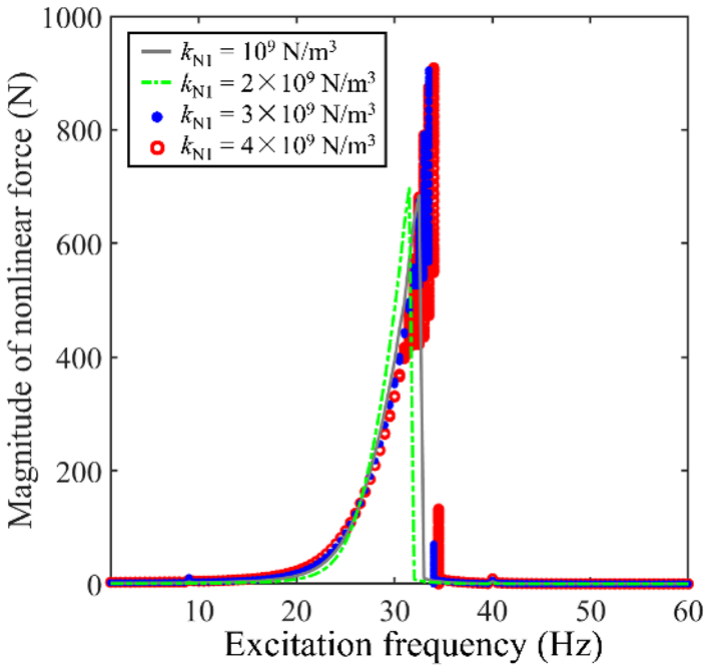
Magnitudes of nonlinear force with the change of *k*_N1_.

Then, to study the potential reason of complex longitudinal vibration responses appearing in Figs. 5 and 7 gives magnitudes of nonlinear force with the change of *k*_N1_. From Fig. 7, with the growth of *k*_N1_, max values at the 1^st^ main resonance area of the nonlinear force gradually increase. When *k*_N1_ increases at a certain value, complex longitudinal vibration responses are also shown in Fig. 7, where the frequency range of complex longitudinal vibration in Fig. 7 perfectly matches those in Fig. 5. Furthermore, it should be noted that the magnitude of nonlinear force jumps at certain frequencies. The above jumping frequencies fit the jumping frequencies in Fig. 5. It can be observed that complex longitudinal vibration responses in Figs. 5 and 7 synchronous occur. The reason for the above phenomenon is that vibration magnitudes of the double-rod system at resonance areas stay in a significant level. Considering the form of nonlinear stiffness acting on internal supports, magnitudes of the nonlinear restoring force is proportion to the displacement of double-rod system. Thus, the nonlinear restoring force acting on the double-rod system stay in a high level under resonance areas. As the excitation frequency runs away from the resonance areas of the double-rod system, vibration magnitude of double-rod system decreases sharply, causing the decrease of nonlinear restoring force. Meanwhile, due to the existence of supporting nonlinear stiffness, magnitude-frequency response curves are no longer symmetric. Thus, magnitude jumping phenomenon can be clearly observed in Fig. 7. Furthermore, the nonlinear support located at Rod 1 only introduces the nonlinear force to the double-rod system. It can be concluded that the growth of nonlinear force is the reason for the occurrence of complex longitudinal vibration responses in Fig. 5.

**Figure 8:**
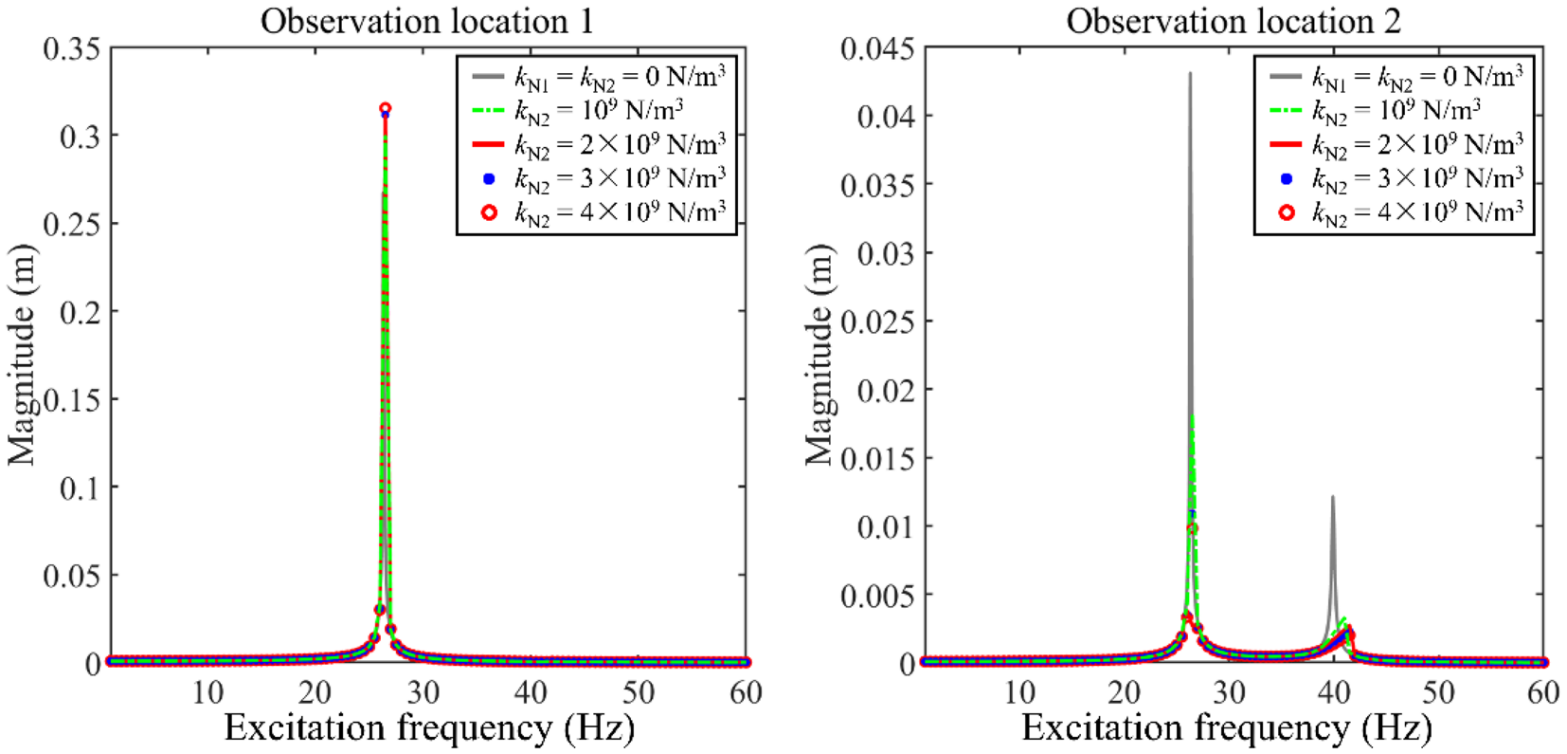
The impact of *k*_N2_ on longitudinal frequency responses.

Figure 8 gives the impact of *k*_N2_ on longitudinal frequency responses. In this numerical example, the longitudinal nonlinear support only installs on Rod 2. According to Fig. 8, the parameter variation of longitudinal nonlinear support which is installed on Rod 2 impacts longitudinal vibration responses greatly for Rod 2. Unfortunately, the variation of *k*_N2_ rarely impacts longitudinal vibration responses for Rod 1. With the growth of *k*_N2_, the 2^nd^ main resonance area gradually shifts to a higher frequency area. The existence of longitudinal nonlinear support suppresses the vibration level of Rod 2. Due to the existence of *k*_N2_, the magnitude jumping phenomenon appears at the 2^nd^ main resonance of Rod 2.

Compressively consider Figs. 5 and 8, it can be found that the longitudinal nonlinear support installed on Rod 1 has a positive impact on vibration reduction for the 1st main resonance area. Meanwhile, the longitudinal nonlinear support installed on Rod 2 positively impacts the vibration reduction of Rod 2. The above phenomenon suggests that the simultaneous installation of longitudinal nonlinear supports on Rod 1 and Rod 2 can effectively reduce the vibration magnitudes of the double-rod system. Therefore, it is of significance to reduce the longitudinal vibration by simultaneously employing longitudinal nonlinear supports.

**Figure 9:**
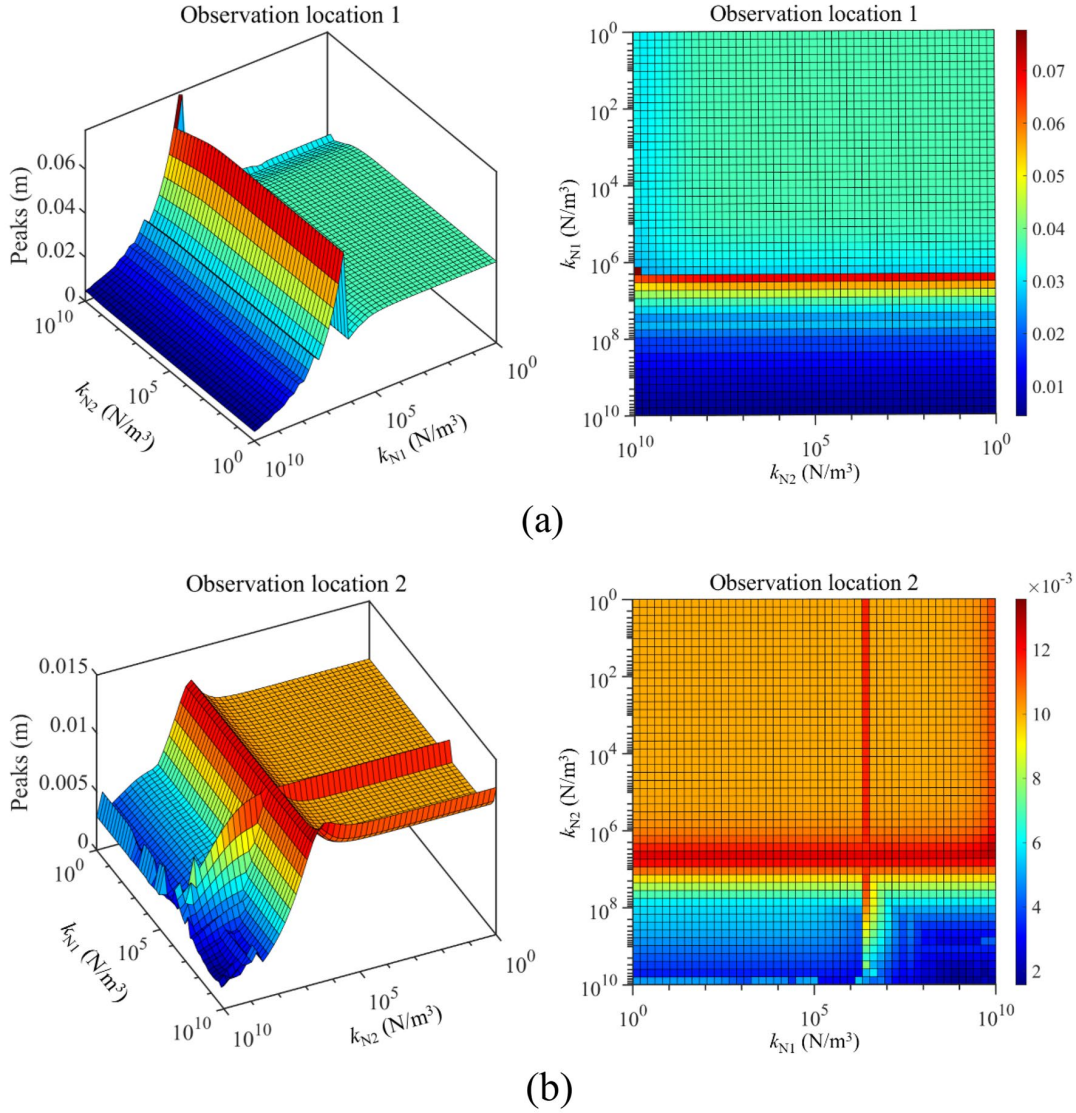
Max values of longitudinal frequency responses under the change of *k*_N1_ and *k*_N2_.

Against this background, Fig. 9 gives the max values of longitudinal frequency responses under the change of *k*_N1_ and *k*_N2_. From Fig. 9, when *k*_N1_ and *k*_N2_ remain at a low level, the impact of longitudinal nonlinear supports on the double-rod system is unobvious. However, changing parameters of longitudinal nonlinear support significantly impacts the max values of frequency response belonging to the double-rod system when the nonlinear stiffness exceeds certain values. The explanation for the above phenomenon is that the magnitudes of nonlinear force are positively correlation with the nonlinear stiffness. Therefore, the nonlinear force stays at a low level under low nonlinear stiffness, causing a limited impact on the maximum values of longitudinal frequency responses belonging to the double-rod system. Furthermore, a reasonable selection of nonlinear stiffness can effectively reduce the vibration of the double-rod system observation location 1 and observation location 2 at the same time.

### Longitudinal vibration responses under determined excitations

On many engineering occasions, the rod system is subjected to excitation caused by power machinery. The power machinery typically works in a determined condition to protect its safety. Against this situation, excitations acting on the rod system present the single-frequency character.

**Figure 10:**
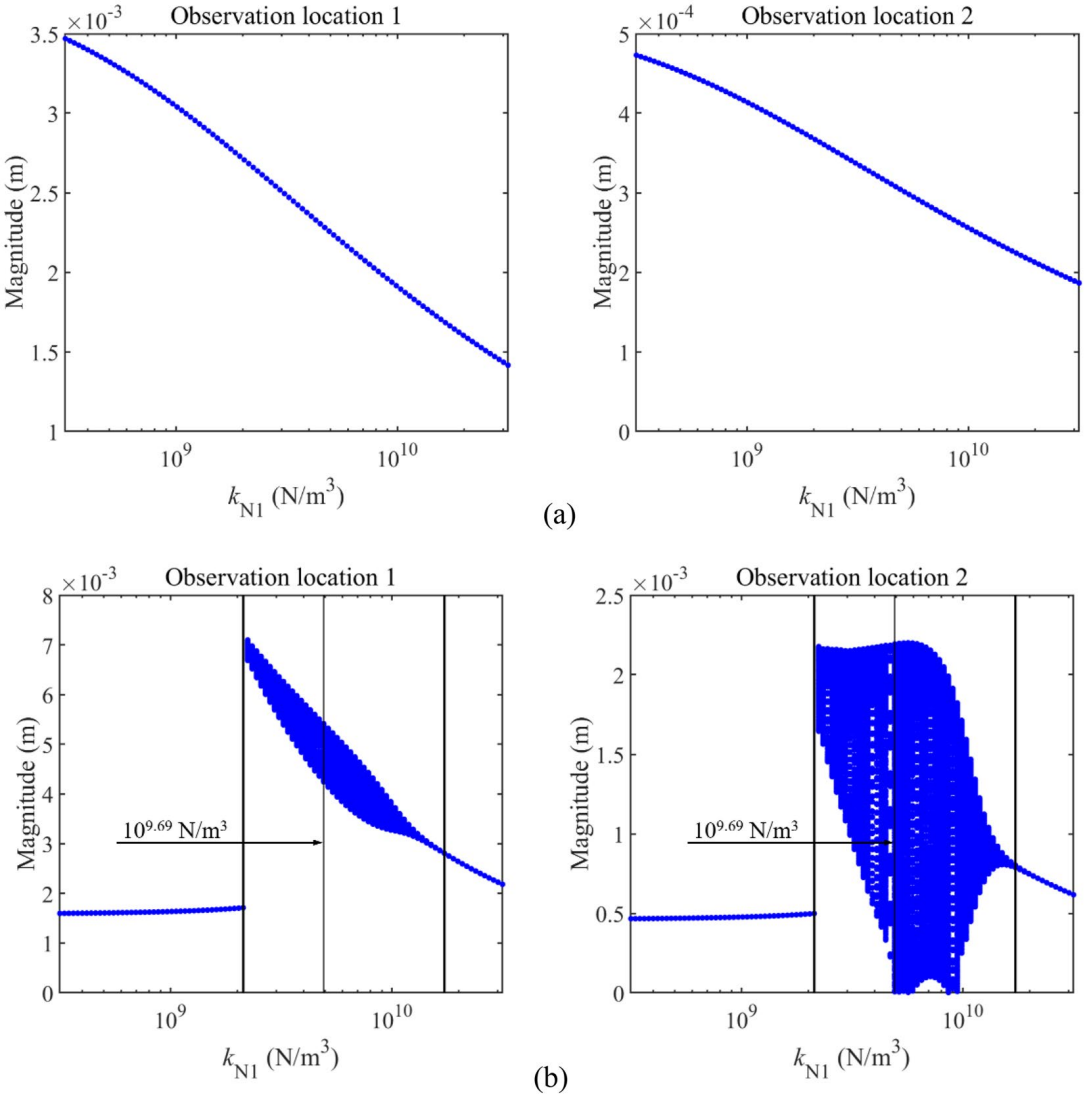
Longitudinal vibration responses under determined excitations impacted by *k*_N1_.

Figure 10 gives the impact of *k*_N1_ on longitudinal vibration responses under determined excitations. For this work, the fundamental natural frequency of Rod 1 is 25.3 Hz while that of Rod 2 is 37.9 Hz. According to the analysis in "Longitudinal frequency responses impacted by longitudinal nonlinear supports" section, for the double-rod system with longitudinal nonlinear supports, 23 Hz is out of the 1st resonance area of double-rod system while 33 Hz is close to the 1^st^ resonance of the double-rod system. To roundly study longitudinal responses of the double-rod system under a single-frequency excitation, excitation frequencies in and out of the resonance areas should be considered. Thus, in this numerical example, frequencies of the harmonic excitation are selected as 23 Hz and 33 Hz. It should be noticed that 23 Hz is out of the 1^st^ main resonance area of the double rod system while 33 Hz is in the 1st main resonance area. From Fig. 10, the change of *k*_N1_ effectively impacts longitudinal vibration responses of the double-rod system under determined excitations. From the aspect of vibration control, the growth of *k*_N1_ is good for reducing the vibration of the double-rod system under 23 Hz. *k*_N1_ staying at a low level is beneficial for the vibration reduction of the double-rod system under 33 Hz. From the aspect of vibration states, the vibration state of the double-rod system always stays in the single-periodic state when the excitation frequency is 23 Hz. However, complex longitudinal vibration responses of the double-rod system appear when the excitation frequency is 33 Hz. With the growth of *k*_N1_, such complex longitudinal vibration responses first appear and then vanish. The reason for the above phenomenon is explained in the following. According to the analysis related to Fig. 5, the growth of *k*_N1_ shifts the 1^st^ main resonance area from a low-frequency area to a high-frequency area. Therefore, with the growth of *k*_N1_, the 1st main resonance area crosses 33 Hz. When the main resonance area contains 33 Hz, the magnitude of longitudinal vibration responses rises sharply, resulting in the increase of the nonlinear force acting on the double-rod system. With the continuous growth of *k*_N1_, the 1st main resonance area is gradually far away from 33 Hz. In the above process, the magnitude of longitudinal vibration responses gradually decreases, resulting in the decrease of the nonlinear force acting on the double-rod system. Due to the variation of nonlinear force, complex longitudinal vibration responses first appear and then vanish.

**Figure 11:**
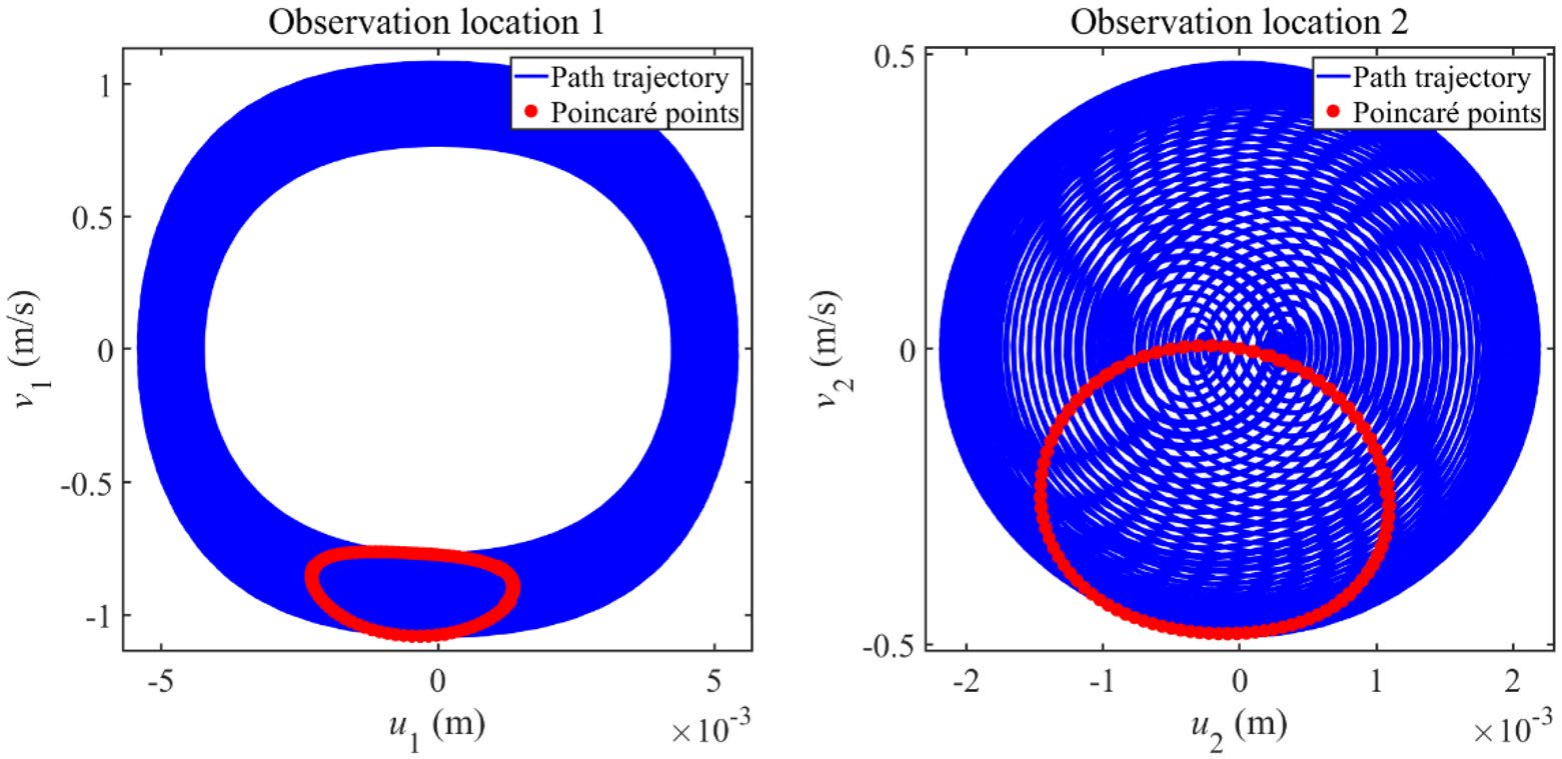
Phase trajectory and Poincaré points of the complex longitudinal vibration responses.

Then, Fig. 11 gives the phase trajectory and Poincaré points of complex longitudinal vibration responses to study their vibration states. From each subfigure in Fig. 11, the phase trajectory remains stable and Poincaré points form an entire closed curve, suggesting that complex longitudinal vibration responses that appeared in Fig. 10 remain in the quasi-periodic vibration state.

**Figure 12:**
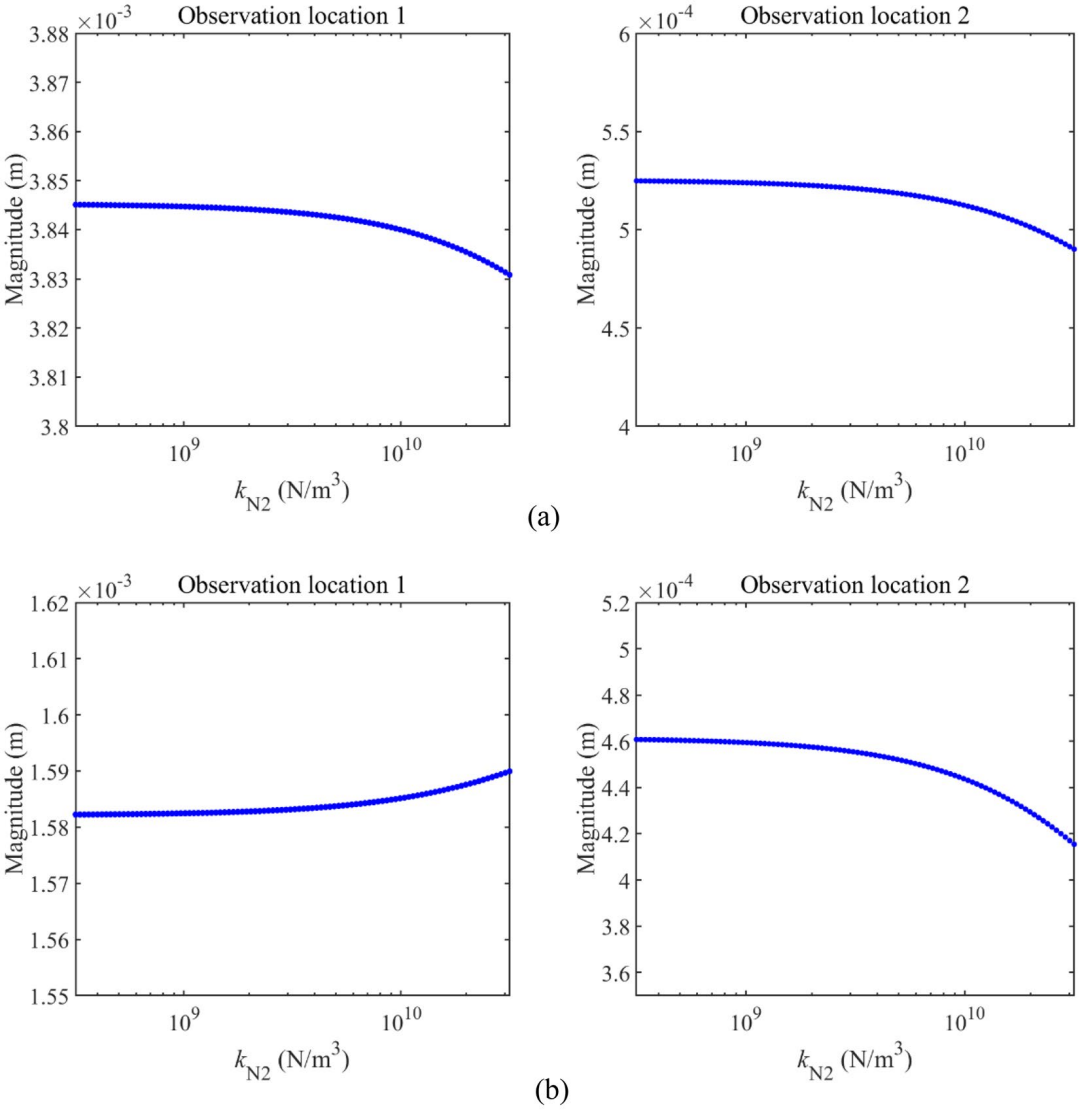
Longitudinal vibration responses under determined excitations impacted by *k*_N2_.

Figure 12 gives the impact of *k*_N2_ on longitudinal vibration responses under determined excitations, where excitation frequencies of the harmonic excitation are also selected as 23 Hz and 33 Hz. From Fig. 12, it can be observed that longitudinal vibration responses of Rod 2 under determined excitations are effectively impacted by the change of *k*_N2_. The growth of *k*_N2_ has a positive impact on the vibration reduction of Rod 2. In contrast, longitudinal vibration responses of Rod 1 under determined excitations are rarely impacted by the change of *k*_N2_, suggesting that it is unwise to control the vibration of Rod 1 by employing *k*_N2_.

Compared the analysis related to Fig. 11 with that related to 12, the impact of *k*_N1_ and *k*_N2_ on the longitudinal vibration responses belonging to the double-rod system is different from each other. Meanwhile, under the excitation frequency located at the 1^st^ main resonance area, longitudinal vibration responses of the double-rod system are more obviously impacted by the change of *k*_N1_ and *k*_N2_. Therefore, it is necessary to study the impact of synchronous change *k*_N1_ and *k*_N2_ on the longitudinal vibration responses of the double-rod system under the excitation frequency located at the main resonance area.

**Figure 13:**
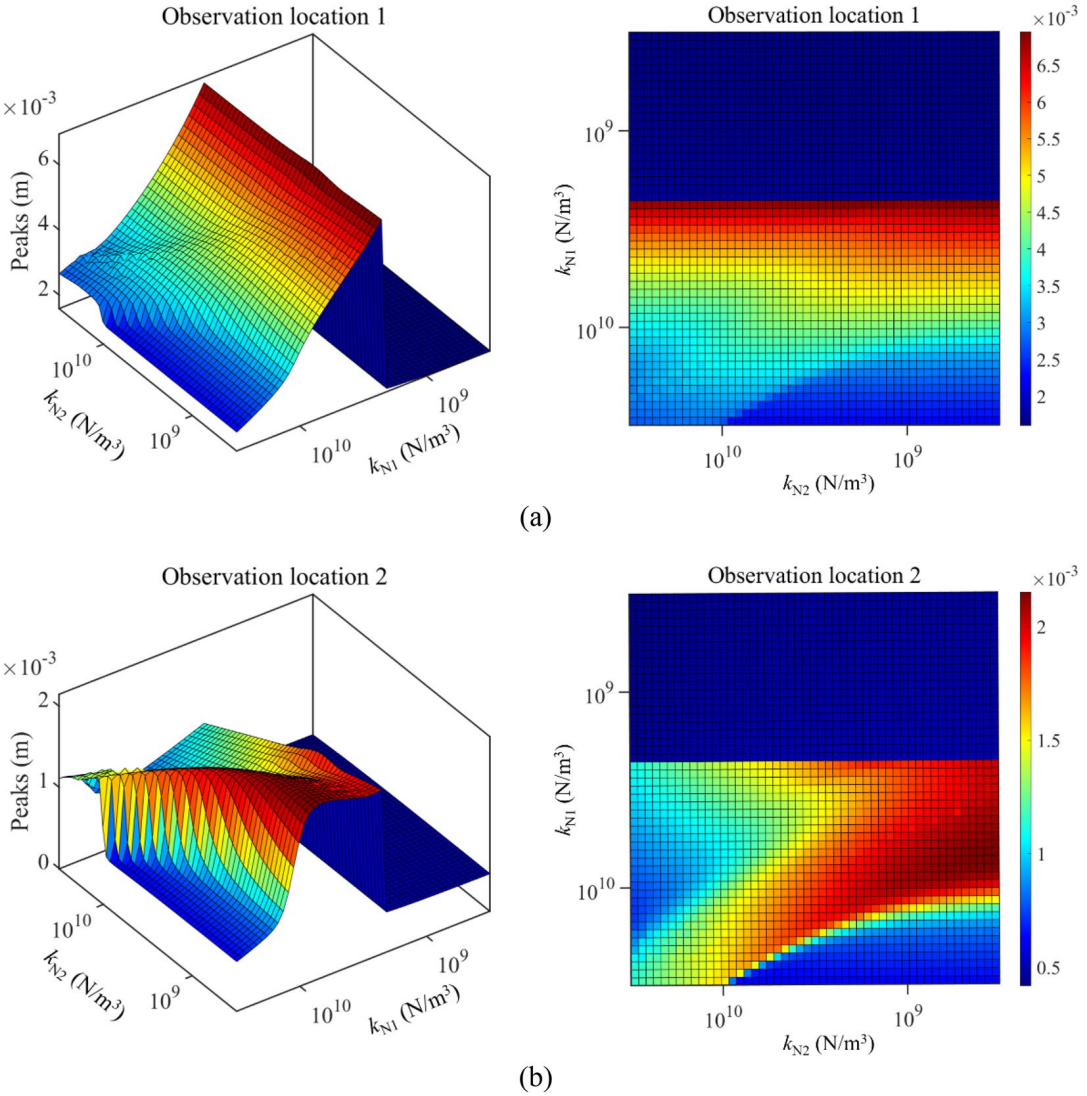
Max values of longitudinal vibration responses with the change of *k*_N1_ and *k*_N2_.

Figure 13 studies the impact of *k*_N1_ and *k*_N2_ on the longitudinal vibration responses of the double-rod system in detail. According to Fig. 13, the impact of *k*_N1_ on the longitudinal vibration responses of the double-rod system has the jumping values. Namely, when *k*_N1_ increases to a certain value, peaks of longitudinal vibration responses increase rapidly. Then, the continuous growth of *k*_N1_ can gradually reduce peaks of the double-rod system. At the same time, it can be found that changing *k*_N2_ also impacts longitudinal vibration responses of the double-rod system when *k*_N1_ exceeds 10^10^ N/m^3^. Furthermore, a reasonable selection of *k*_N1_ and *k*_N2_ is positive for the vibration reduction of the double-rod system.

## Conclusion

This work introduces a longitudinal vibration prediction model for a double-rod system equipped with longitudinal nonlinear supports, with the supports installed on each sub-rod. We solve the longitudinal vibration responses of the double-rod system using the GTM. We discuss in detail the influence of longitudinal nonlinear supports, installed on each sub-rod, on the double-rod system’s vibration responses. Moreover, we explain the causes behind complex longitudinal vibration responses. The conclusions of this work are summarized as follows,

1. We propose and validate a longitudinal vibration prediction model for a double-rod system with longitudinal nonlinear supports. For the parameters selected in this study, a 1-term truncation number ensures the stability of the prediction model.
2. Longitudinal nonlinear supports on each sub-rod significantly affect the double-rod system’s vibration responses. Adjusting the parameters of these supports alters the vibration state and magnitude. Installing longitudinal nonlinear supports on both Rod 1 and Rod 2 effectively reduces vibration.
3. Complex longitudinal vibration responses are more readily induced by altering the parameters of the longitudinal nonlinear support on Rod 1. Careful selection of parameters for the supports on both Rod 1 and Rod 2 is beneficial for reducing vibrations in the double-rod system.
4. Overall, this study systematically explores the impact of nonlinear supports on the longitudinal vibration control of the double-rod system, enhancing the potential for applying nonlinear supports to control shafting system vibrations in marine engineering.

### Supplementary Information

Supplementary Information.
