# Supplementary material for: A study of analyzing longitudinal dynamic behavior of a double-rod system with longitudinal nonlinear supports
Source: Sci Rep. 2024 Apr 7;14:8126. doi: 10.1038/s41598-024-58986-9 (PMC10999428; doi:10.1038/s41598-024-58986-9)
Supplement: Supplementary file 1 — Supplementary Information. [file 41598_2024_58986_MOESM1_ESM.docx]

# APPENDIX A

$T_{\text{Rod1}}=\frac{1}{2}\rho_{1}S_{1}\int_{0}^{L_{1}} \left(\frac{\partial u_{1}}{\partial t}\right)^{2}\text{d}x_{1}$ (A-1)

$T_{\text{Rod2}}=\frac{1}{2}\rho_{2}S_{2}\int_{0}^{L_{2}} \left(\frac{\partial u_{2}}{\partial t}\right)^{2}\text{d}x_{2}$ (A-2)

$V_{\text{Rod1}}=\frac{1}{2}E_{1}S_{1}\int_{0}^{L_{1}} \left(\frac{\partial u_{1}}{\partial x_{1}}\right)^{2}\text{d}x_{1}$ (A-3)

$V_{\text{Rod2}}=\frac{1}{2}E_{2}S_{2}\int_{0}^{L_{2}} \left(\frac{\partial u_{2}}{\partial x_{2}}\right)^{2}\text{d}x_{2}$ (A-4)

$V_{\text{Boundary1}}=\frac{1}{2}k_{\text{L1}}u_{1}^{2}\left(0,t\right)+\frac{1}{2}k_{\text{R1}}u_{1}^{2}\left(L_{1},t\right)$ (A-5)

$V_{\text{Boundary2}}=\frac{1}{2}k_{\text{L2}}u_{2}^{2}\left(0,t\right)+\frac{1}{2}k_{\text{R2}}u_{2}^{2}\left(L_{2},t\right)$ (A-6)

$V_{\text{LN1}}=\int_{0}^{L_{1}} \delta\left(x_{1}-x_{\text{N1}}\right)\frac{1}{4}k_{\text{N1}}u_{1}^{4}\text{d}x_{1}$ (A-7)

$V_{\text{LN2}}=\int_{0}^{L_{2}} \delta\left(x_{2}-x_{\text{N2}}\right)\frac{1}{4}k_{\text{N2}}u_{2}^{4}\text{d}x_{2}$ (A-8)

$V_{\text{LE}}=\int_{0}^{L_{2}} \int_{0}^{L_{1}} \delta\left(x_{1}-L_{1}\right)\delta\left(x_{2}\right)\frac{1}{2}k_{\text{E}}\left(u_{1}-u_{2}\right)^{2}\text{d}x_{1}\text{d}x_{2}$ (A-9)

$\delta W_{\text{E}}=\int_{0}^{L_{1}} \delta\left(x_{1}\right)F_{\text{E}}\sin\left(\omega t\right)\delta u_{1}\text{d}x_{1}$ (A-10)

$\delta W_{\text{LE}}=-\int_{0}^{L_{2}} \int_{0}^{L_{1}} \delta\left(x_{1}-L_{1}\right)\delta\left(x_{2}\right)C_{\text{E}}\left[\frac{\partial u_{1}}{\partial t}-\frac{\partial u_{2}}{\partial t}\right]\delta\left(u_{1}-u_{2}\right)\text{d}x_{1}\text{d}x_{2}$ (A-11)

# APPENDIX B

Governing equations of rods are derived by putting Eqs. (B-1) to (B-11) into Eq. (4).

$\int_{t_{1}}^{t_{2}} \delta T_{\text{Rod1}}\text{d}t=-\int_{t_{1}}^{t_{2}} \int_{0}^{L_{1}} \rho_{1}S_{1}\frac{\partial^{2}u_{1}}{\partial t^{2}}\text{d}x_{1}\delta u_{1}\text{d}t$ (B-1)

$\int_{t_{1}}^{t_{2}} \delta T_{\text{Rod2}}\text{d}t=-\int_{t_{1}}^{t_{2}} \int_{0}^{L_{2}} \rho_{2}S_{2}\frac{\partial^{2}u_{2}}{\partial t^{2}}\text{d}x_{2}\delta u_{2}\text{d}t$ (B-2)

$\int_{t_{1}}^{t_{2}} \delta V_{\text{Rod1}}dt=\int_{t_{1}}^{t_{2}} \left(\left.E_{1}S_{1}\frac{\partial u_{1}}{\partial x_{1}}\right|_{x_{1}=L_{1}}-\left.E_{1}S_{1}\frac{\partial u_{1}}{\partial x_{1}}\right|_{x_{1}=0}\right)\delta u_{1}\text{d}t-\int_{t_{1}}^{t_{2}} \int_{0}^{L_{1}} E_{1}S_{1}\frac{\partial^{2}u_{1}}{\partial x_{1}^{2}}\text{d}x_{1}\delta u_{1}\text{d}t$ (B-3)

$\int_{t_{1}}^{t_{2}} \delta V_{\text{Rod2}}dt=\int_{t_{1}}^{t_{2}} \left(\left.E_{2}S_{2}\frac{\partial u_{2}}{\partial x_{2}}\right|_{x_{2}=L_{2}}-\left.E_{2}S_{2}\frac{\partial u_{2}}{\partial x_{2}}\right|_{x_{2}=0}\right)\delta u_{2}\text{d}t-\int_{t_{1}}^{t_{2}} \int_{0}^{L_{2}} E_{2}S_{2}\frac{\partial^{2}u_{2}}{\partial x_{2}^{2}}\text{d}x_{2}\delta u_{2}\text{d}t$(B-4)

$\int_{t_{1}}^{t_{2}} \delta V_{\text{Boundary1}}\text{d}t=\int_{t_{1}}^{t_{2}} \left[k_{\text{L1}}u_{1}\left(0,t\right)+k_{\text{R1}}u_{1}\left(L_{1},t\right)\right]\delta u_{1}\text{d}t$ (B-5)

$\int_{t_{1}}^{t_{2}} \delta V_{\text{Boundary2}}\text{d}t=\int_{t_{1}}^{t_{2}} \left[k_{\text{L2}}u_{2}\left(0,t\right)+k_{\text{R2}}u_{2}\left(L_{2},t\right)\right]\delta u_{2}\text{d}t$ (B-6)

$\begin{matrix} \int_{t_{1}}^{t_{2}} \delta V_{\text{LE}}\text{d}t=\int_{t_{1}}^{t_{2}} \delta\left(x_{1}-L_{1}\right)k_{\text{E}}\left[u_{1}-u_{2}\left(0,t\right)\right]\delta u_{1}\text{d}t \\ \begin{matrix} & & & +\int_{t_{1}}^{t_{2}} \delta\left(x_{2}\right)k_{\text{E}}\left[u_{2}-u_{1}\left(L_{1},t\right)\right]\delta u_{2}\text{d}t \end{matrix} \end{matrix}$ (B-7)

$\int_{t_{1}}^{t_{2}} \delta V_{\text{LN1}}\text{d}t=\int_{t_{1}}^{t_{2}} \int_{0}^{L_{1}} \delta\left(x_{1}-x_{\text{N1}}\right)k_{\text{N1}}u_{1}^{3}\delta u_{1}\text{d}x_{1}\text{d}t$ (B-8)

$\int_{t_{1}}^{t_{2}} \delta V_{\text{LN2}}\text{d}t=\int_{t_{1}}^{t_{2}} \int_{0}^{L_{2}} \delta\left(x_{2}-x_{\text{N2}}\right)k_{\text{N2}}u_{2}^{3}\delta u_{2}\text{d}x_{2}\text{d}t$ (B-9)

$\int_{t_{1}}^{t_{2}} \delta W_{\text{E}}\text{d}t=\int_{0}^{L_{1}} \delta\left(x_{1}\right)F_{\text{E}}\sin\left(\omega t\right)\text{d}x_{1}\delta u_{1}\text{d}t$ (B-10)

$\begin{matrix} \int_{t_{1}}^{t_{2}} \delta W_{\text{LE}}\text{d}t=-\int_{t_{1}}^{t_{2}} \int_{0}^{L_{2}} \int_{0}^{L_{1}} \delta\left(x_{1}-L_{1}\right)\delta\left(x_{2}\right)C_{\text{E}}\left[\frac{\partial u_{1}}{\partial t}-\frac{\partial u_{2}}{\partial t}\right]\text{d}x_{1}\text{d}x_{2}\delta u_{1}\text{d}t \\ \begin{matrix} & & & -\int_{t_{1}}^{t_{2}} \int_{0}^{L_{2}} \int_{0}^{L_{1}} \delta\left(x_{1}-L_{1}\right)\delta\left(x_{2}\right)C_{\text{E}}\left[\frac{\partial u_{2}}{\partial t}-\frac{\partial u_{1}}{\partial t}\right]\text{d}x_{1}\text{d}x_{2}\delta u_{2}\text{d}t \end{matrix} \end{matrix}$ (B-11)

# APPENDIX C

In LM, the Lagrange term of the double-rod system with longitudinal nonlinear supports is derived as,

$L_{\text{System}}=T_{\text{System}}-V_{\text{System}}+W_{\text{System}}$ (C-1)

To simply establish the Lagrange function, the longitudinal vibrational displacements of the double-rod system with longitudinal nonlinear supports are reformed into specific terms, which are outlined as follows,

 (C-2)

and

 (C-3)

where the form terms are shown in Eqs. (C-2) and (C-3) are listed as,

 (C-4)

 (C-5)

$\mathbf{q}_{1}=\left[\begin{matrix} q_{11} & ... & q_{1n} & ... & q_{\text{1N}} \end{matrix}\right]^{\text{T}}$ (C-6)

and

$\mathbf{q}_{2}=\left[\begin{matrix} q_{21} & ... & q_{2m} & ... & q_{\text{2M}} \end{matrix}\right]^{\text{T}}$ (C-7)

By substituting Eqs. (C-2) and (C-3) into Eq. (C-1) and proceed with the subsequent step, namely

 (C-8)

The Lagrange function of the double-rod system with longitudinal nonlinear supports can be established. By solving the Lagrange function, the longitudinal dynamic behavior of the double-rod system can be obtained.

# APPENDIX D

In HBM, the longitudinal vibration displacements of the double-rod system are expanded as Eq. (7). Unknown time terms of the longitudinal vibration displacements are assumed as,

$q_{1n}\left(t\right)=B_{1n}\sin\left(\omega t\right)+B_{2n}\cos\left(\omega t\right)+C_{1n}\sin\left(3\omega t\right)+C_{2n}\cos\left(3\omega t\right)$ (D-1)

and

$q_{2m}\left(t\right)=E_{1m}\sin\left(\omega t\right)+E_{2m}\cos\left(\omega t\right)+G_{1m}\sin\left(3\omega t\right)+G_{2m}\cos\left(3\omega t\right)$ (D-2)

where *B*_1_*_n_*, *B*_2_*_n_*, *C*_1_*_n_*, *C*_2_*_n_*, *E*_1_*_m_*, *E*_2_*_m_*, *G*_1_*_m_*, and *G*_2_*_m_* are the unknown coefficients. Substituting Eqs. (D-1) and (D-2) into Eq. (8), the functions related to unknown coefficients can be then obtained by arranging terms related to sin(*ωt*), cos(*ωt*), sin(3*ωt*), and cos(3*ωt*). Longitudinal dynamic behavior of the double-rod system with longitudinal nonlinear supports can be obtained by solving the above functions.
